# Medical students’ awareness and perception of national health examinations

**DOI:** 10.7603/s40681-014-0021-2

**Published:** 2014-08-25

**Authors:** Lii-Tzu Wu, Tsuei-Yuan Lai, Chiu-Shong Liu, Cheng-Chun Lee, Cheng-Chieh Lin, Meei-Ling Horng

**Affiliations:** 1 School of Medicine, College of Medicine, China Medical University, Taichung, Taiwan; 2 Department and Graduate Institute of Special Education, National Changhua University of Education, Changhua, Taiwan; 3 Department of Medical Research, China Medical University Hospital, Taichung, Taiwan; 4 Department of Family Medicine, China medical University Hospital, Taichung, Taiwan; 5 Department of Neurology, China Medical University Hospital, Taichung, Taiwan; 6 Center for General Education, China Medical University,, 91 Hsueh-Shih Road, Taichung, Taiwan

**Keywords:** Medical education;, Medical students;, National health examination;, T-test and ANOVA;, Self-directed learning

## Abstract

Key ingredients for upgrading health care include bolstering and appraising professional medical education. Health examination as a crucial element of health care that we must incorporate into medical education. This research evaluates medical students’ awareness of national health examinations. Two surveys, focused on health examination knowledge and perspective, were conducted for first- to fourthyear medical students, results analyzed by descriptive statistics, t-test and ANOVA. Research subjects scored maximum 11 (of possible 15): i.e., 76.2% accuracy for health examination knowledge questions and held positive views on seven (58%) perspective-related questions. Self-directed learning courses do provide a positive effect on students’ learning. Respondents’ varying backgrounds had insignificant impact on overall results, but in-depth analysis for each individual question does reveal differences among several backgrounds. Medical students’ overall awareness level for health examination is above average in comparison to the general public. This research result can provide a basis to improve the related professional programs, courses and teachings or used as a reference for modifications on future classes. The above observations were discussed based on the medical education system in Taiwan.

## 1. Introduction

New challenges arise everyday in a fast-changing society, with medical departments and agencies worldwide forced to adapt via new and immediate solutions. In recent years, apart from stress on professional skills in medical education reforms, there is increased focus on humanity as a mission for social health care [1,2]. Taiwanese medical education has thus agreed on doctors' professional development as vital to relevant medical reforms: (1) integrating fundamental and clinical courses, (2) nurture of students’ self-directed learning, (3) team learning, (4) health treatment for humanity, (5) enhancing clinical skills and early clinical exposure, (6) facing fundamental and community health care [3]. Medical education has gradually shifted its focus to integrating with practical application and connecting seamlessly with medical education in advanced nations, making it pivotal to integrate fundamental and clinical courses while reconstructing content of medical education in a holistic way.

Since emphasis on citizens’ health is clearly defined, medical departments worldwide have evolved comprehensive care systems for their citizens [4,5]. Taiwan, with its people steadily accumulating economic means and their lives ever more stable, has begun to realize the importance of performing regular health examinations. Due to widespread use of computers and rapid growth of Internet, citizens’ knowledge of health care resources likewise escalates [6]. This raises the importance of analyzing and interpreting health exam results; further problems emerge if citizens lack accurate understanding thereof. Hence, appropriate knowledge, such as interpretation, further medical counseling, etc., must be given to health examination subjects to assist them to view health care and medicine from a prevention standpoint. Holistic care forms a concept for viewing individual as the core basis, family as center and community as parameter to provide medical care and prevention health care services [7].

The practical application of the holistic health care can only be achieved with a quality holistic medical education program [8]. Hence all medical schools should practice holistic medical education, implement diverse program design, hoping this will allow future medical students to become well qualified doctors [9]. Initial experimental design is survey with items related to Taiwan’s Ministry of Health & Welfare (http://www.nhi.gov.tw), providing health examination policies so as to ascertain whether first- to fourth-year medical students are well equipped with knowledge and perspective relevant to citizens’ health issues, as references for current medical curricula and implement diverse program design that medical education needs to function.

## 2. Methods

Through literature review, this research identified key factors in health examination, survey instrument designed in accordance with these key components. Expert reviews and pre-test were conducted before actual experiment. The following sections will elaborate on research samples, tools, and data processing.

### 2.1. Research subjects

Population used in this research consists of students from a medical college located in central Taiwan in year 2011. Samples in pre-test were selected by purposive sampling: 11 first-year plus 10 second-, third- and fourth-year students, with a total of 41 testing samples. Formal samples were chosen by convenience sampling, 360 students selected from the population as research subjects; deletion of incomplete surveys left 335 ones.

### 2.2. Research instrument

As per the aim of this study and through literature review and team discussion, first version of “Medical Students’ Awareness Level towards Health Examination” was drafted. Survey was divided into two parts: (1) subjects’ demographics, composed of seven questions; and (2) knowledge and perspective on health examinations, with 15 questions related to knowledge and 14 to perspective, for a total of 29 questions. To enhance quality of the survey content, expert opinions on appropriateness of content, diction and categorization were gathered. The survey was then edited in accordance with suggestions provided by the experts.

Pre-test sampled 41 students. After surveys were collected, the scale for each individual question was analyzed to determine critical ratio (CR) and significance level. Questions not reaching significance (CR>3 or p>0.05) were deleted to safeguard distinctiveness between scale options, survey analyzed for consistency, and questions with Cronbach *α* value too low removed to enhance overall Cronbach *α* value to 0.62. Per the reliability and validity analysis results, final version of survey instrument consisted of 15 questions on knowledge and 12 on perspective. Overall reliability and validity of instrument was above average.

### 2.3. Data analysis

After collecting all survey results, ineffective surveys were removed, statistical methods determined by characteristics of the data as well as items warranting analysis by statistical software, SPSS 18.0 for Windows. Methods such as descriptive analysis, t-test, one-way ANOVA etc. evaluated resulting data.

## 3. Results

### 3.1. Subjects’ demographics

Of 360 initial surveys, 354 were collected (98% return rate), with 335 effective (95%). Nine basic items of information were collected about survey subjects. Ratio of male to female was 3:2, roughly equal numbers from each grade, parents’ education level either college or university, fathers’ occupations chiefly business-oriented, mothers’ occupation varied across other categories, most subjects underwent health examination once or twice in the past five years for a gamut of reasons and samples with volunteer hospital experience is about 3:2 (Table 1).

### 3.2. Health examination knowledge and perspective

Survey items were modeled after health examination checklist items promoted by Taiwan’s Ministry of Health and Welfare. Format used *yes* or *no* answers for each question, correct answer yielding one point and incorrect answer zero: i.e., maximum score of 15 points, with total average points utilized for analysis. Higher score meant greater awareness (Table 2-A). Subjects overall averaged above 11 (correct percentage 76.2%). For the correct percentage of each individual question (except Questions 2, 10, and 14 with less than half the samples answered correctly), the rest were above 50%. Nine had correct percentage above 90%; total scores indicate awareness of health examination promoted by Taiwan’s Ministry of Health of Welfare as sufficient.

Internet search divulged frequently asked questions on health examinations as survey items to test medical students’ understanding of health examination concepts. Survey questions were answered on a scale of 1-5: 1=strongly agree and 5=strongly disagree. Scores should be calculated as percentages ranging from agree/no comment/disagree (Table 2-B). Among twelve items, medical students have more positive perspective for seven on health examinations (results over 40%: Question 2 (40.3%), Question 3 (55.8%), Question 4 (77%) highest of all, Question 5 (39.3%), Question 7 (51%), Question 8 (53.7%), and Question 1 (49.3%). More students answered incorrectly on Questions 1, 6, and 12. This last had the lowest percentage: i.e., uncertainty about categories offered in a regular health examination (10.5%). In the self-evaluation section, skill level to read blood sample data and determine ECG normality is quite low, as portended by Questions 9 (24.9%) and 10 (14.9%)

**Table 1:** Demographic data of medical students.

		N=335	
items	groups	no. of students	percentage(%)
Gender	(1) male (2) female	197 138	58.8 41.2
Class year	(1) freshman (2) sophomore (3) junior (4) senior	83 77 96 79	24.8 23.0 28.7 23.6
Educational Background (Father)	(1) elementary (2) junior high (3) senior high (4) college or university (5) graduate	3 17 69 186 73	0.9 6.1 20.6 50.1 21.8
Types of Job (Father)	(1) business (2) military or government (3) freelance (4) agriculture and fishery (5) other	130 84 39 4 74	38.3 25.1 11.6 1.2 22.1
Educational Background (Mother)	(1) elementary (2) junior high (3) senior high (4) college or university (5) graduate	6 20 111 158 38	1.8 6.0 33.1 47.2 11.3
Types of Job (Mother)	(1) business (2) military or government (3) freelance (4) agriculture and fishery (5) other	70 97 51 2 110	21.2 29.4 15.5 0.6 32.8
Number of Health Examinations Conducted in the Last Five Years	(1) 0 (2) one (3) two (4) three (5) four (6) above five	26 118 115 43 10 17	7.8 35.2 34.3 12.8 3.0 5.1
Reasons for Health Examination	(1) family medical history (2) personal illness (3) insurance (4) other	4 36 29 200	1.2 10.7 8.7 59.7
Volunteer Experience in Hospital	(1) yes (2) no	206 127	61.5 37.9

**Table 2 - A.** Research subjects’ medical knowledge (based on examination promoted by ministry of health and welfare). Score.

			N=335	
Question		Avg.	SD	Above mean
1.	Female over the age of 30 can receive a free cervical smear once per year.	0.61	0.49	✓
2.	Ministry of Health and Welfare provide free health examination once every third year for citizens over age 40.	0.36	0.48	
3.	Only those beyond age 40 need to receive regular health examinations.	0.93	0.25	✓
4.	People under age 40 who do not experience discomfort need no health examination.	0.95	0.21	✓
5.	If results are normal for this health examination, then no further examination is necessary.	0.97	0.18	✓
6.	Apart from minor sickness, health examinations can also prevent major health issues (such as cancer, diabetes or cardiovascular disease).	0.91	0.28	✓
7.	Health examinations can detect all the sickness symptoms regardless of how serious it is.	0.92	0.27	✓
8.	Health examinations are only provided by major hospitals.	0.91	0.29	✓
9.	Health examination results not affected by female subjects conducted during their period.	0.90	0.30	✓
10.	Ministry of Health & Welfare provide free yearly oral health check for citizens over age 18 with smoking or chewing tobacco habits.	0.31	0.46	
11.	Health examinations should be taken once biennially before age 30 and annually between 30-50; early start means better effect.	0.79	0.41	✓
12.	Health examination items should be decided based on person health conditions, family health history and economic situations.	0.92	0.27	✓
13.	Kids under the age of 7 does not need health examinations.	0.92	0.27	✓
14.	Ministry of Health & Welfare provides citizens aged 50-60, free colorectal cancer screen biennially.	0.45	0.50	
15.	Ministry of Health & Welfare provides female aged 50-69 free biennial breast radiology.	0.56	0.50	✓
Total Average Score		11.43	2.12	

**B.** Research subjects’ health examinations perspective scoring percentage.

Question		% of agree/ have no comment / disagree	Missing value
1.	Average consensus: sufficient for health examinations to include x-ray, blood test, and ECG.	49.8/21.5/26.9	1.8
2.	Serum liver function check is normal, indicating no chronic hepatitis or cirrhosis.	28.7/28.1/40.3	3.0
3.	Health examinations all conducted by department physicians.	15.5/26.9/55.8	1.8
4.	Preventative health care should cover the psychology and physiological including symptoms of the body and the mind.	77/14.9/6.6	1.5
5.	It is sufficient for patients with hepatitis B to perform blood checks every three to six months to ensure the surface antigen and liver functions.	22.4/36.4/39.3	2.1
6.	Examinations items such as endoscopy, x-ray 、upper gastrointestinal examination and stomach ultrasonic can be conducted by technical personnel and the doctors only need to perform the readings.	50.5/23.6/29.6	2.7
7.	During health examinations, check items such as ENT and ophthalmology does not need to be conducted by specialized doctors, it is sufficient for family physicians or medical department physicians to perform the examinations.	20.9/36.3/51	1.8
8.	Health examinations can be conducted even when subjects are not feeling well.	53.7/24.8/19.7	1.8
9.	I can read the basic blood sample data to determine whether it is normal.	24.5/31/42.1	2.4
10.	I can judgmentally determine whether the ECG results is normal.	14.9/26.9/57	1.2
11.	I can provide health care knowledge, health education, and promote health knowledge related to health examinations.	49.3/34.6/14.6	1.5
12.	An average health examination consists of three items to prevent professional illness, adult health care prevention and health examination for the elderly.	54/34/10.5	1.5

Underlined percentage numbers indicate perspective positive

Abbreviations: Avg.: Average; SD: Standard deviation

**Table 3:** ANOVA awareness level towards health examination for different grade level’s medical students.

								N=335		
Scale	Class Yrs.	Pop.	Avg.	SD	ANOVA					
					Source of difference	Deviation from mean	Degree of freedom	Mean square	F value	Comp.
Health	1	83	11.41	2.05	Inter group	2.97	3	2.34	0.52	
Exam	2	77	11.23	2.07	Intra group	1498.99	331	4.53		
Knowledge	3	96	11.29	2.42	total	1501.96	334			
	4	79	11.56	1.92						
Health	1	83	35.22	4.29	Inter group	61.06	3	27.19	0.68	
Exam	2	77	34.91	7.17	Intra group	13198.47	331	39.98		
Perspective	3	96	34.44	6.84	total	13259.52	334			
	4	79	34.09	6.54						
Knowledge	1	83	0.23	0.42	Inter group	1.95	3	0.65	3.06*	3>1
10	2	77	0.30	0.46	Intra group	70.14	331	0.21		
	3	96	0.43	0.50	total	72.10	334			
	4	79	0.28	0.45						
Perspective	1	83	3.42	0.89	Inter group	6.92	3	2.31	2.70*	
5	2	74	3.22	0.86	Intra group	276.59	324	0.85		
	3	93	3.11	0.91	total	283.51	327			
	4	78	3.04	1.02						
Perspective	1	82	3.22	0.89	Inter group	15.19	3	5.06	4.73**	2>4
9	2	73	3.66	1.00	Intra group	345.79	323	1.07		
	3	95	3.26	1.05	total	360.98	326			
	4	77	3.04	1.07						
Perspective	1	83	2.61	0.99	Inter group	8.83	3	2.95	3.94**	1>4
12	2	74	2.58	0.89	Intra group	243.52	326	0.74		
	3	95	2.52	0.74	total	252.35	329			
	4	78	2.19	0.84						

*p<0.05

Abbreviations: Yrs.: Years; Pop.: Population; Avg.: Average; SD: Standard deviation; Comp.: Comparison

### 3.3. Effect of gender and hospital volunteer experiences on awareness level towards health examinations

Using gender and hospital volunteer experiences as independent and survey answers as dependent variables, independent sample t-test gauges health examination knowledge and perspective as a result of students’ background. Results indicate no significant difference in total and individual score for each question.

### 3.4. Medical students’ awareness level toward health examination reflected by different backgrounds

Using grade level of students, parents’ educational level and/or occupation, plus number of health examinations within the last five years as independent and survey answers as dependent variables for one-way ANOVA, overall results indicated that differences in background had no direct correlation with scoring in knowledge and perspective. Analysis of each question reveals differences relating to backgrounds.

#### 3.4.1. Medical students of different grade levels

Four items scored substantial differences between grade levels, using Scheffé comparison, substantial differences noted for three. On Question 10, juniors outscored freshmen. On perspective Question 9 relating to reading blood sample data, agreement level for seniors was substantially higher than for sophomores. On perspective Question 12, freshmen also showed a higher agreement level than seniors (Table 3).

#### 3.4.2. Father’s job and mother’s education

Two items in the knowledge section yielded sharp contrast: by Scheffé comparison, substantial difference appeared in Question 4, fathers’ job business-related higher than for military- or government-related. (Table 4-A) For comparison between samples with mother’s educational level varying, no difference noted between total scores, but five items attained substantial differences; by Scheffé comparison, substantial differences were noted with two questions. Knowledge Question 5 relates to if results are normal for this health examination, then no further examination is necessary, agreement among those with mothers’ education at elementary school is lower than those with mothers’ educational level at junior/senior high or above college. Perspective Question 1 deals with understanding of health examination, samples with mothers’ educational level at senior high scored higher than those with mothers’ educational level at junior high. (Table 4-B)

#### 3.4.3. Varying reasons for health examinations

Results show no difference in total scores, but two questions reached substantial differences for each group of samples, the same results shown by Scheffé comparison. Knowledge question 4 asks “if a person is under 40 and experiences no discomfort, then no medical check is needed.” Knowledge question 9 asks whether “period will impact health check results.” Samples with health examination reasons are scored significantly higher than insurance-related. (Table 5)

## 4. Discussion

Medical students’ understanding and caring for the health care system constitutes a critical topic for medical education reform. Health examination is one fundamental concept for health care: letting citizens grasp the importance of health examinations as one key policy promoted by the national government[5]. While many articles relate to physical health assessment [10-12], few validation studies published relate to how fundamental medical education can enhance medical students’ humanist qualities, to combine what they learn in class and apply to surrounding environment, to implement holistic health care.

Subjects scored more than 0.5 (12/15 takes about 80%) on 12 of 15 questions in the health care knowledge section, this indicates that most medical students have sufficient knowledge toward health care. In the knowledge section, Question 2 has average score of 0.36 and Question 14 0.45; this reflects that students are less familiar with free examination items. Question 10 has the lowest among all questions (average 0.31); further analyses indicate grade level, fathers’ occupation, and mothers’ educational level significantly differ. Scheffé comparison avers grade level as significant: juniors averaging highest at 0.43 and freshmen 0.23 for lowest. Question 10 is on health examination item related to Taiwanese citizens’ smoking or chewing tobacco, lead cause of oral cancer[13]. To enhance college and university level health consciousness, the Education Ministry’s 2011 policy encouraged school to proactively prevent damage caused by smoking. This policy trained sophomores by problem-based learning (PBL), such that they understand effect on health due to smoking via case study and discussion. Having researched smoking, they scored higher on knowledge items. Questions 2, 10 and 14 have scores less than 0.5 and this is due to the differences in age and differences in health examination needs. Evidence-based medicine has been proven that many diseases relate directly with age bracket [14,15], for which many differences arise f r om h ealth examination items, a topic and theme meriting emphasis and discussion in related classes.

**Table 4 - A:** ANOVA awareness level towards health examination for medical students with regard to father’s job.

							N=335			
Scale	Fathers’ job	Pop.	Avg.	SD	ANOVA					
					Source of difference	Deviation from mean	Degree of freedom	Mean square	F value	Comp.
Health	1	130	11.62	1.90	Inter group	9.84	4	2.45	0.56	
Exam	2	84	11.42	2.33	Intra group	1442.01	326	4.42		
Knowledge	3	39	11.38	2.26	total	1451.83	330			
	4	4	10.50	1.73						
	5	74	11.27	2.11						
Health	1	130	34.35	7.09	Inter group	102.68	4	25.67	0.64	
Exam	2	84	35.17	5.65	Intra group	13071.11	326	40.10		
Perspective	3	39	33.87	6.71	total	13173.79	330			
	4	4	37.50	3.70						
	5	74	35.07	5.46						
Knowledge	1	130	0.99	0.87	Inter group	0.52	4	0.13	3.05*	1>2
4	2	84	0.89	0.31	Inter group	13. 80	326	0.04		
	3	39	0.95	0.22	total	14.32				
	4	4	1.00	0						
Knowledge	1	130	0.37	0.48	Inter group	2.35	4	0.59	2.79*	
10	2	84	0.21	0.41	Intra group	68.60	326	0.21		
	3	39	0.44	0.50	total	70.95	330			
	4	4	0	0						
	5	74	0.27	0.45						

Note 1 = Business, 2 = Military or government, 3 = Freelance, 4 = Agriculture and fishery, 5 = Other

Abbreviations: Pop: Population; Avg.: Average; SD: Standard deviation; Comp.: Comparison

**B.** ANOVA awareness level towards health examination for medical students with regard to mother’s educational level.

							N=335			
Scale	Mothers’ EDU BG	Pop.	Avg.	SD	ANOVA					
					Source of difference	Deviation from mean	Degree of freedom	Mean square	F value	Comp.
Health	1	6	11.60	2.07	Inter group	9.84	4	2.45	0.56	
Exam	2	20	11.41	2.40	Intra group	1442.01	328	4.42		
Knowledge	3	111	11.57	1.93	total	1451.83	332			
	4	158	10.50	1.73						
	5	38	11.27	2.11						
Health	1	6	32.50	2.26	Inter group	43.55	4	10.89	0.27	
Exam	2	20	34.40	6.99	Intra group	13156.78	328	40.11		
Perspective	3	111	34.54	7.90	total	13200.26	332			
	4	158	34.94	5.26						
	5	38	34.53	5.20						
Knowledge 5	1	6	0.37	0.48	Inter group	2.35	4	0.59	2.79*	
	2	20	0.21	0.41	Inter group	69.18	328	0.21		
	3	111	0.44	0.50	total	71.52	332			
	4	158	0	0						
	5	38	0.27	0.45						
Knowledge 11	1	6	0.67	0.56	Inter group	2.08	4	0.52	3.23*	
	2	20	0.55	0.51	Inter group	52.63	328	0.16		
	3	111	0.83	0.38	total	54.70	332			
	4	158	0.83	0.38						
	5	38	0.68	0.47						
Knowledge 13	1	6	0.83	0.41	Inter group	0.76	4	0.19	2.67*	
	2	20	0.75	0.44	Inter group	23.22	328	0.07		
	3	111	0.93	0.26	total	23.97	332			
	4	158	0.93	0.26						
	5	38	0.97	0.16						
Perspective 1	1	6	2.67	1.21	Inter group	16.77	4	4.19	3.26*	3>2
	2	20	1.90	0.97	Inter group	414.65	322	1.29		
	3	111	2.86	1.19	total	431.42	326			
	4	158	2.72	1.12						
	5	38	2.53	1.11						

*p<0.05, **p<0.01

Note 1=Elementary, 2=Junior High, 3=Senior High, 4=College or University, 5=Graduate Abbreviations: ED: Educational; BG: Background; Pop.: Population; Avg.: Average; SD: Standard deviation; Comp.: Comparison

**Table 5:** ANOVA awareness level towards health examination for medical students with regard to reasons for health exam.

							N=335			
Scale	Reasons of exam	Pop.	Avg.	SD	ANOVA					
					Source of difference	Deviation from mean	Degree of freedom	Mean square	F value	Comp.
Health	1	4	11.00	1.41	Inter group	15.06	3	5.02	1.15	
Exam	2	36	11.92	1.75	Intra group	1153.24	265	4.35		
Knowledge	3	29	11.02	2.92	total	1168.30	268			
	4	200	11.32	2.01						
Health	1	4	35.00	3.56	Inter group	121. 67	3	27.19	0.68	
Exam	2	36	34.94	5.93	Intra group	9671.65	265	39.98		
Perspective	3	29	32.69	6.81	total	9793.32	268			
	4	200	34.84	5.97						
Knowledge	1	4	1.00	0	Inter group	0.54	3	0.18	3.74*	2>3
4	2	36	1.00	0	Intra group	12.73	265	0.05		4>3
	3	29	0.83	0.38	total	13.27	268			
	4	200	0.96	0.21						
Knowledge	1	4	1.00	0	Inter group	1.32	3	0.44	5.23**	2>3
9	2	36	1.00	0	Inter group	22.17	265	0.08		2>3
	3	29	0.72	0.45	total	23.48	268			
	4	200	0.91	0.29						

*p0.05, ** *p*<0.01

Note 1=Family medical history, 2=Personal illness, 3=Insurance, 4=Other

Abbreviations: Pop.: Population; Avg.: Average; SD: Standard deviation; Comp.: Comparison

Seven questions are answered more positively from “health examination” perspective (7/12=58%), indicating that subjects have average understanding of health examination concepts. Of these, Questions 9-11 test whether students possess fundamental skills after taking related classes; perspective Questions 9 and 11 are more positive: Question 9 relates to courses for juniors and seniors to read blood sample data. Differential analysis proves seniors’ agreement level starkly higher than for sophomores, showing that case studies offered within schools’ integrated curriculum for juniors and seniors enhances learning; 49.3% feel they can provide information related to health care knowledge and promoting health education (Question 11). This may be due to all respondents coming from medical school, and related courses in service learning t aught since 2009 [16]. Such an idea was noted by Dewey, American educationalist, in the 1960s: “learn by doing,” integrates service and classroom courses to transform society’s resources as pedagogical material [17]. It mainly uses health-related themes in activities for subjects ranging from grade school students to community elders. Respondents have experiences in this realm, and these are required freshman courses. Differential analysis for this question reflects that freshmen who have taken this class have higher self-recognition than students in other grades. Items answered wrongly include Question 1, addressing professional medical courses, among which is common latent cancers, a task entailing professional examination assistance [18]. Question 6 relates to medical responsibility: examination items performed by professionals. Question 10 is on clinical practice, ability to read ECG, perhaps because this task requires more experience; students generally feel diffident here. Question 12 assesses categories offered in health examination that encompass more than three categories; substantial discrepancy arises in its differential analysis. Comparison afterwards reflects freshmen recognizing more such categories than seniors; reasons merit exploration. Sections linked to health examinations with more misconceptions include fundamentals and clinical practice; this lends reference for design of material.

## 6. Conclusion

This study concludes that medical students possess fundamental knowledge towards health examination policies promoted by Taiwan’s Ministry of Health and Welfare albeit only an average health examination-related perspective; overall awareness of knowledge and perspective on health examination is deemed above average. Self-conducted courses such as problem-based learning (PBL) and service learning will imbue a much stronger impression on related topics. Some differences exist in medical students of varying backgrounds, worth future research to gain further understanding. The above results can enhance related professional courses and teachings, as well as reconstruct programs’ content quality in future medical education reform discussions to view reflect societal needs. Most effort now must focus on incorporating awareness of national examination policies within medical fundamental profession and clinical practice to build holistic healthcare learning environment that renders well-qualified doctors to ensure national health.

## Acknowledgements

We wish to thank anonymous interns and residents from China Medical University Hospital for helping modify our survey.

## Declaration of Interest:

Authors declare no conflicts of interest for this work.
